# Mapping of initiatives to increase membership in mutual health organizations in Benin

**DOI:** 10.1186/1475-9276-11-74

**Published:** 2012-12-05

**Authors:** Anne-Marie Turcotte-Tremblay, Slim Haddad, Ismaïlou Yacoubou, Pierre Fournier

**Affiliations:** 1 University of Montreal Hospital Research Centre, 3875 Saint-Urbain Street, Room 5-01, Montreal, Quebec, H2W 1V1, CANADA; 2 Department of Social and Preventive Medicine, Faculty of Medicine, University of Montreal, 1420 Mont-Royal Boulevard, Montreal, Quebec, H2V 4P3, CANADA; 3 Centre d'étude et d'Appui Technique aux Institutions de Micro assurance Santé (A.I.M.S.), Parakou, 02BP866, Republic of Benin

**Keywords:** Mutual health organization, Membership, Enrolment, Health insurance, Benin

## Abstract

**Introduction:**

Mutual health organizations (MHO) have been implemented across Africa to increase access to healthcare and improve financial protection. Despite efforts to develop MHOs, low levels of both initial enrolment and membership renewals continue to threaten their financial viability. The purpose of this study was to map initiatives implemented to increase the pool of MHO members in Benin.

**Methods:**

A multiple case study was conducted to assess MHOs supported by five major promoters in Benin. Three months of fieldwork resulted in 23 semi-structured interviews and two focus groups with MHO promoters, technicians, elected members, and health professionals affiliated with the MHOs. Fifteen non-structured interviews provided additional information and a valuable source of triangulation.

**Results:**

MHOs have adopted a wide range of initiatives targeting different entry points and involving a variety of stakeholders. Initiatives have included new types of collective health insurance packages and efforts to raise awareness by going door-to-door and organizing health education workshops. Different types of partnerships have been established to strengthen relationships with healthcare professionals and political leaders. However, the selection and implementation of these initiatives have been limited by insufficient financial and human resources.

**Conclusions:**

The study highlights the importance of prioritizing sustainable strategies to increase MHO membership. No single MHO initiative has been able to resolve the issue of low membership on its own. If combined, existing initiatives could provide a comprehensive and inclusive approach that would target all entry points and include key stakeholders such as household decision-makers, MHO elected members, healthcare professionals, community leaders, governmental authorities, medical advisors, and promoters. There is a need to evaluate empirically the implementation of these interventions. Mechanisms to promote dialogue between MHO stakeholders would be useful to devise innovative strategies, avoid repeating unsuccessful ones, and develop a coordinated plan to promote MHOs.

## Introduction

One hundred million people are pushed into poverty every year because of direct payments for healthcare services [1]. According to the 2010 World Health Report [1], community health insurance plans can play a useful role by redirecting some of the direct payments into prepaid pools, thereby expanding protection against the financial risk of ill health and helping people understand the benefits of being insured. Over the past few years, these experiences have proliferated across Francophone African countries, under the rubric of “mutual health organizations” (MHO). In 2003, it was estimated that there were 622 MHOs in 11 Francophone African countries [2]. While insurance plans can take many different forms [3], studies have consistently shown a low level of coverage and limited membership in sub-Saharan Africa [4]. Most MHOs reach less than 10% of their target population [5], and their membership in sub-Saharan Africa rarely exceeds more than a few hundred beneficiaries. As in all insurance mechanisms, the pool size of insured individuals plays a determinant role in MHOs’ predictability of spending and financial equilibrium [6] and thus greatly affects their viability. To survive, most MHOs have required the financial and technical assistance of promoters supported by States and cooperation agencies.

The body of knowledge on factors that contribute to low levels of initial enrolment and membership renewal is well established. The primary causes of low membership are multifactorial and generally include a combination of poor quality of healthcare, poverty, distrust in MHO representatives, dissatisfaction with the insurance packages, lack of available information on MHOs, and cultural beliefs with respect to diseases [7-13]. MHO representatives are now dedicating considerable effort to developing innovative strategies that address the root causes of low membership.

To this day, there is a gap in knowledge about what interventions could increase MHO membership in West Africa. Some researchers have recommended that MHOs should seek to better understand consumers’ preferences and incorporate these into the design of insurance plans [11,14]. Churchill and Cohen [15] proposed that MHOs should conduct marketing campaigns to increase people’s awareness and understanding of simple insurance products. These authors have pointed out that microinsurance advertisements typically convey notions of protection, solidarity, optimism, and trust. Marketing campaigns in countries such as Bangladesh have relied on a wide range of communicators (e.g. healthcare workers, social workers, teachers, and government officials) and have used street theatre and pictorial representations to target specific groups such as illiterates. Churchill and Cohen concluded that greater attention, creativity and resources are required to promote MHOs. Finally, some authors have proposed that MHOs should seek support from central and local governments [16-18].

To our knowledge, there is no published review on best practices for increasing MHO membership. Past studies have sought to identify factors that reduce enrolment, but without exploring in depth how these have been addressed in different organizations and contexts. There is a need to gain a better understanding of these initiatives, including analyses of the local stakeholders’ perception of their benefits and disadvantages within different contexts. The present study was conducted in Benin to map and review initiatives adopted by MHOs to increase membership levels. Specifically, the aim of the study was to document (1) the process that led to the selection and implementation of initiatives to increase membership, (2) the challenges and facilitating factors associated with the implementation of these initiatives, and (3) how these initiatives influenced membership levels in comparison with MHO actors’ expectations.

## Methodology

### Design

Conducted in 2008–2009, this study was approved by the ethics committee of the University of Montreal and an *ad hoc* committee organized in Benin. We developed a multiple case-study design to capture the diversity of MHOs’ approaches for dealing with low membership levels [19]. The five major MHO promoters in Benin were selected, each constituting a case. The first author conducted three months of extensive fieldwork in Benin to participate in local life, visit MHO-related organizations, and understand how membership issues were perceived and addressed in each case. The design involved conducting interviews, examining documentary materials, and organizing focus groups to stimulate dialogue between local stakeholders. For reasons of length, this paper will focus on the data obtained during the interviews.

### Cases selected

In an environment in which most of the population has no access to healthcare insurance, MHOs have proliferated in Benin since 2001 with the support of bilateral (France, United States, Belgium, Switzerland, and Denmark) and multilateral (ILO) agencies. In 2008, there were approximately 135 MHOs in the country, most supported by one of the five promoters included in this study (see Table 1 for detailed descriptions of each case).

Case 1:*Centre International de Développement et de Recherche (CIDR).* Between 1994 and 2008, this French non-governmental organization (NGO) implemented MHOs in Benin with the financial assistance of the Swiss government. These MHOs are organized into a complex structure. Each MHO covers numerous villages located within an administrative subdivision called an *arrondissement* (borough). In each village, members are divided into four or five groups to promote solidarity and help collect premiums. Each group has a delegate who sits on the MHO’s administrative committee. MHOs in the same health district then create what is called an *inter-MHO*. The inter-MHO’s main responsibility is to negotiate with district hospitals. All MHOs are part of the *Réseau alliance santé* (RAS), or health network alliance. The RAS aims to (1) reinforce management competencies, (2) maintain a security fund from which money can be loaned to MHOs when finances are low, (3) act as a second-level insurance fund to help MHOs develop their activities and (4) establish development objectives with MHOs. To fund these services, MHOs allocate a percentage of their premiums to the RAS. In 2007, the RAS was composed of 30 MHOs covering 37,117 members in good standing (i.e., whose premium payments were up-to-date).

Case 2:*Programme d’Appui aux Mutuelles de Santé en Afrique (PROMUSAF)*. This program was created in 1998 by a Belgian NGO called *Solidarité mondiale* funded by the *Fonds belge de survie*. Also established in Burkina Faso, Senegal, and Mali, PROMUSAF supported 25 MHOs in Benin in 2007, covering 2 353 members in good standing. One feature of PROMUSAF is that it also offers micro-credit to its members.

Case 3:*Projet Intégré de Santé Familiale* (PISAF). Launched in 2006, PISAF is a project supported by the United States Agency for International Development (USAID). In 2007, MHOs affiliated with PISAF covered 2 823 members in good standing. In 2009, PISAF supported 28 MHOs in Benin and was preparing to create 17 new ones.

Case 4:*Association pour le Développement de la Mutualité Agricole au Bénin (ADMAB)*. Created in 1996, ADMAB is a Beninese organization that receives financial support from France. ADMAB developed a two-pronged system of health insurance, in which a portion of members’ contributions is placed in a personal health savings plan to cover small risks and another portion goes into a mutual health insurance pool for larger risks. It also offers complementary programs such as health education, agricultural support, and free schooling for poor children. In 2007, ADMAB covered approximately 2 750 members in good standing. By 2009, it was supporting 13 MHOs across Benin.

Case 5:International Labour Organization/Strategies and Tools against Social Exclusion and Poverty (ILO-STEP). ILO-STEP began activities in Benin in 2002. In 2009, it supported six MHOs, including the MHO created by the Ministry of Labour and Public Service, called the *Mutuelle de sécurité sociale du Bénin*. In 2007, these mutual health organizations covered 11 808 members in good standing.

**Table 1:** Descriptions of the five (5) major MHO promoters included in the study

**Promoter**	**CIDR**	**PROMUSAF**	**PISAF**	**ADMAB**	**ILO-STEP**
Starting year for MHOs	1995	1999	2006 (previously PHR*plus*)	1996	2003
# of MHOs	30	25	28	13	6
Type	Third-party payment	Third-party payment	Third-party payment	Combines personal health savings for small risks with third-party payment for big risks	Third-party payment
Members in good standing (premiums paid up to date)	*Insurance for Families* = 20 118	2 353	2 823	2 750	11 808
	*Student Insurance* =16 434				
	Other products: 565				
Premiums (variations exist for each MHOs)	*Insurance for Families*: Fees based on family size	200 F CFA per person per month	200 F CFA per person per month (annual payment is encouraged)	Three options available between 15 000 and 25 000 F CFA per family per year	For the one (1) MHO affiliated with the State: 600 F CFA per person per month
	*Student Insurance:* 350 F CFA per student per year			65% of the premium is for individual health savings, 30% for the solidarity fund (risk sharing), 5% for management. In addition, members must replenish any health savings paid out during the previous year.	
	*Maternity without Risks*: 350 F CFA per individual in the village per year				
Method of enrolment	*Insurance for Families*:	Voluntary individual enrolment	Voluntary family enrolment	Voluntary family enrolment	For the MHO affiliated with the State: individual enrolment and automatic enrolment of members of groups that joined the MHO
	Voluntary family enrolment			Families of up to 11 members.	
	In some cases, families must join a small subgroup of MHO members in their neighbourhood to enrol				
	*All collective products*:				
	Automatic enrolment of members of groups that joined the MHO				
Coverage	Depends on MHOs, but *Insurance for Families* usually covers 75% of fees for ambulance, prenatal consultations, hospitalizations, urgent surgeries and complicated deliveries in hospitals, deliveries and observation in local health centres	Covers 75% of services in health care centres and 60% of services in hospital.	Covers from 75% to 80% of services offered in the government’s Minimum Package of Activities in health care centres and hospital care	Health savings: Covers healthcare services offered in peripheral healthcare centres. Solidarity (risk sharing): Covers, completely or partially, fees for evacuation to a hospital	Depends on the MHO
	*Student Insurance*: Covers 100% of ambulance fees and hospitalizations, including medication and surgeries, for problems arising during school hours				For the MHO affiliated with the government: Covers 70% of health fees
	*Maternity without Risks*: Covers deliveries and prenatal consultations of women in the village				

### Participants within cases

We used a snowball approach to select participants to be interviewed. A key informant identified the first series of participants affiliated with the five different MHO promoters, who in turn helped identify other series of potential participants. Participants were selected based on the relevance of their relationship to MHOs, the possibility of providing new information, and their level of accessibility. Following the diversification principle, we included participants with a variety of intrinsic qualities, such as associations with different settings, regions, and levels of urbanization [20]. They were contacted by telephone or in person. No financial compensation was offered for their participation, and all gave their free and informed consent. No one contacted refused to participate in the study.

### Instruments

We developed a semi-structured interview guide containing 10 open-ended questions (see Appendix A) to obtain participants’ perceptions on four main issues: (1) MHO membership levels; (2) the causes of low levels of enrolment and membership renewal; (3) the initiatives implemented to increase membership, and their advantages, limitations, and perceived outcomes; and (4) the future of MHOs in Benin. Experts reviewed the semi-structured interview guide to ensure construct validity. We also pilot tested the guide to refine the questions and vocabulary. Data from the pilot testing were not used in this study. The interviews lasted one hour on average.

**Figure 1:**
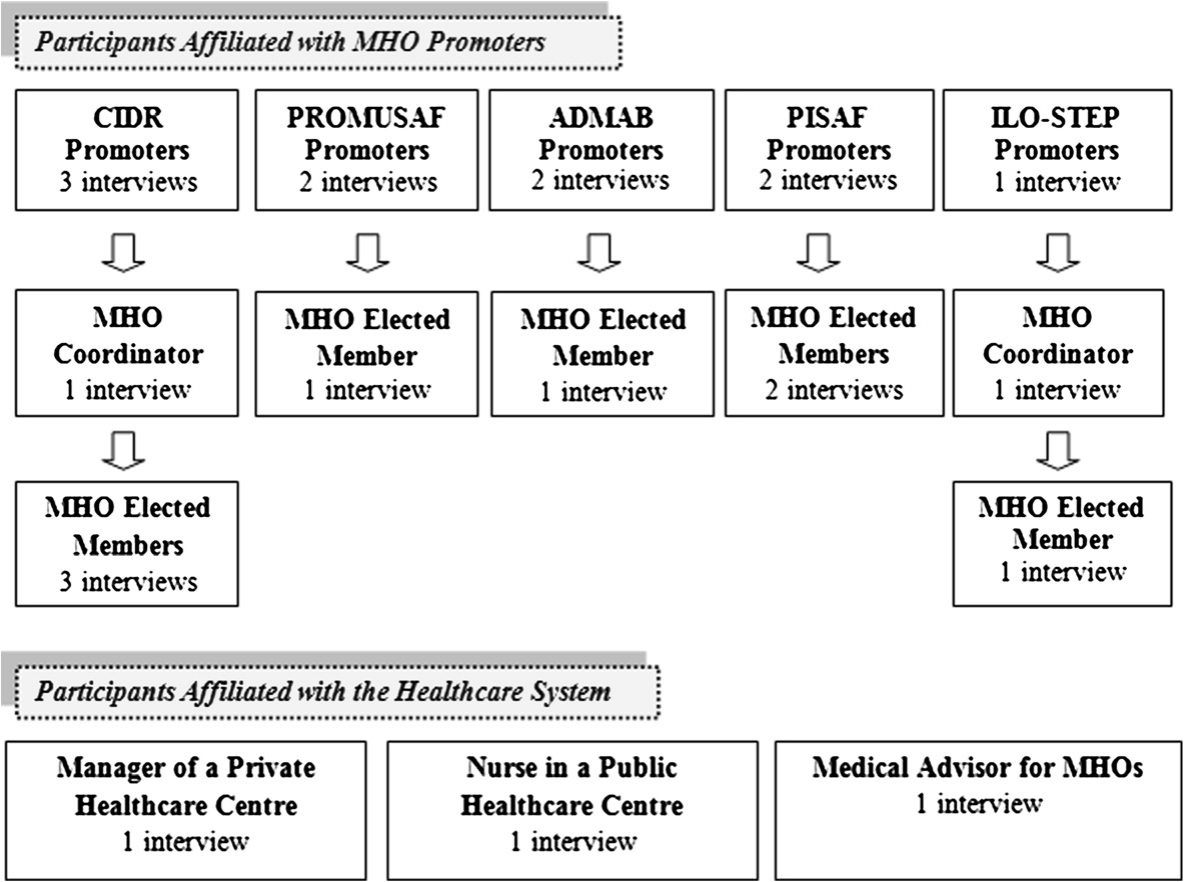
Graphic representation of the semi-structured interviews.

Twenty-three participants underwent semi-structured interviews (see Figure 1): 10 promoter representatives, two coordinators, one technical assistant, eight elected members, one healthcare worker, and one health centre manager. We also conducted non-structured interviews, for triangulation, with one medical doctor, eight elected members, and six healthcare workers.

### Data analysis

The semi-structured interviews were recorded and transcribed by a professional stenographer, and the transcripts were analyzed using QDA Miner. Codes were determined through a mixed process, i.e., certain codes were identified in advance while others were added during the analysis [21]. Content analysis was performed on data collected from the non-structured interviews. Applying the principle of saturation, we stopped collecting data when interviews, and observations no longer provided information that was sufficiently different to justify continuing data collection.

## Results

A wide variety of initiatives, presented below, have been implemented to increase enrolment and/or membership renewal. Approaches have varied greatly from one promoter to another. Although MHOs supported by the same promoter remained independent from one another, they tended to adopt similar initiatives. An additional file to this article summarizes the initiatives adopted to increase enrolment and/or membership renewal in Benin [see Additional file 1: Table S2. Mapping of Initiatives to Increase Membership in MHOs].

### Facilitate payments

*“There are very hard periods where people do not have any money at all, not even to eat…”* (Participant)

Some participants affiliated with MHOs supported by PISAF, PROMUSAF, and ILO-STEP reported adopting monthly payments because they found it easier to pay small portions of membership fees progressively. The main challenge of this approach was the workload it imposed on elected MHO members, who had to collect payments every month. This highly time-consuming task also incurred high transportation costs for elected members. Moreover, in some cases, it was difficult for health facilities to keep track from month to month of which patients were covered. To overcome these limitations, MHOs affiliated with the RAS opted for annual fees collected during periods of incoming revenues. Advocates of this annual fee approach argued that it was adapted to the reality of local rural populations because major decisions on income allocation are taken when harvests are sold. Participants also argued that annual premiums require much less work for elected members responsible for collecting membership fees.

Some MHOs offered individual premiums to make it easier for single persons to gather the money to enrol. This type of membership may be more adapted to the individualist mentality slowly emerging in cities. The negative repercussion was that sick individuals tended to join more than healthy ones, thus reducing risk-sharing. In contrast, MHOs affiliated with the RAS rejected individual memberships because it led to higher costs for large families. Instead, they opted for family membership, in which different group prices are set for different categories of family size and larger families pay less per person than smaller families. This encourages mutual aid among families of different sizes.

Participants reported that because cash was not always readily available in rural areas, some MHOs allowed members to pay in kind (e.g., crops). These MHOs would stock the crops temporarily and sell them when prices increased on the international market. Participants reported that this measure presents certain limitations, including the risk of the market price falling before products are sold and the fact that most MHOs do not own large and safe storage areas, such that animals may destroy or eat the crops.

ADMAB developed agricultural cooperatives to finance MHOs. Members of nearby villages collectively cultivate fields. However, it was reported that access to the field can sometimes be difficult as some villages are a few kilometres away and transportation costs limit members’ participation.

A minority of MHOs have managed to obtain subsidies from local authorities. The Ministry of Labour and Public Service is providing degressive funding to the *Mutuelle de sécurité sociale du Bénin* until the MHO reaches a predetermined level of beneficiaries. In return, the MHO has pledged to achieve growth objectives. In addition, local authorities in Nikki, a rural commune, provided 5 million F CFA to subsidize the RAS’ collective products for pregnant women and students. Similarly, local authorities in Sinendé offered 800 000 F CFA to PISAF MHOs to support their implementation. However, these subsidies were not used to specifically target the poor or destitute.

Participants called for more subsidies to finance MHOs:

“*If every year, the local authorities gave 800 000 F CFA…we could at least get a manager in order to get some stability over there!*” (Participant)

### Improve the quality of healthcare and the patient–healthcare worker interpersonal relationship

*“I would say that the percentage of members who leave [MHOs] because of the negative behaviour of health professionals is 30%”.* (Participant)

According to participants, many healthcare workers provided inferior health services to MHO members by being rude to them and sometimes withholding medication. Participants explained that this behaviour was mainly due to the fact that healthcare workers could not sell services or medication for their own profit because costs were pre-determined with the MHO. Moreover, under-the-table payments were not possible because payments were generally made directly from the MHO’s account to the health facility’s account. This cashless system went against the personal interests of some health professionals.

Approaches to improve relationships with healthcare workers and increase the availability of medicine varied from one provider to another. All promoters but one had strengthened relationships with healthcare workers by establishing contracts that specified payment modalities, insurance coverage, conflict resolution procedures, medication availability, and workload for healthcare workers. Contracting was adopted because, according to most participants, informal agreements had negative consequences on membership levels.

Urban MHOs affiliated with the RAS worked with the Network for Coordinated Care (NCC). Established in 2002, the NCC was an alliance of eight health facilities that set rules for collaboration with MHOs. Meetings between the NCC and elected MHO members were organized every three months, with negotiations resulting in lower healthcare prices for MHOs. One benefit of the NCC was that, because it included a variety of public and private health facilities, MHO members could obtain services and technical support from whichever facilities had them available. Healthcare workers could refer patients to other NCC facilities for specific care or medication not available at their own facility.

*“With this association, there is self-regulation. We feel the situation is more organized compared to places that don’t have it.… To be part of that, [healthcare centres] must respect certain principles, for example, pre-established tariffs”.* (Participant)

*“The NCC organized a seminar to train the staff who have first contact with patients.… If this first person doesn’t greet sick people properly, it brings down the whole system, even the good parts.… After the seminar, it was three months before we received another complaint”.* (Participant)

Adopting a different approach, PISAF initiated a partnership to involve healthcare workers directly in promoting MHOs. Healthcare workers held workshops to devise action plans. They also received training to reinforce their competence to offer high-quality care. Teams composed of healthcare workers, elected members, and members of community health management committees organized activities every month to raise awareness. Teams of elected members and healthcare workers met monthly to go door-to-door or mobilized small groups to increase awareness. This partnership was reported to have strengthened the credibility of MHOs. Elected members stated that their collaboration with healthcare workers was difficult at first but improved over time.

*“At first, healthcare workers did not accept that we establish this type of dialogue. But over time, they understood that MHOs are important and they accepted the collaboration.… Elected members were scared to get closer to healthcare workers but now they trust them. They work together”.* (Participant)

A few participants were concerned that directly involving healthcare workers in the MHOs’ development would be risky, given that healthcare workers have, in some cases, illegitimately prescribed themselves medication at the MHO’s expense.

Participants from ADMAB, adopting a co-development approach, stressed that it was difficult to develop MHOs without simultaneously supporting health facilities. ADMAB therefore provided health facilities with medication, equipment, ambulances, sources of water, and access to electricity. In return, those health facilities offered a 10% to 25% discount on healthcare costs. Some participants felt this approach positively influenced the quality of care and the relationship between MHO members and healthcare workers. Because of these resource infusions, healthcare workers now perceived providing care to MHO members as an opportunity to improve their working conditions. However, participants recognized that this motivation to collaborate with MHOs could be jeopardized if the promoter discontinued its support.

Participants reported that technical support from medical advisors improved the collaboration between healthcare workers and MHOs. With their knowledge and expertise in the medical field, medical advisors facilitated negotiations between healthcare workers and MHO leadership. Although there was great demand for more support from medical advisors, their high fees put a strain on available resources thereby limiting the frequency of their services.

*“The presence of a medical advisor is very important because healthcare workers are uncontrollable.… He can really sensitize and negotiate with healthcare workers”.* (Participant)

To improve healthcare services offered to members, the RAS identified criteria that health facilities had to satisfy in order to collaborate with MHOs. Participants described how medical advisors evaluated the health facilities’ technical equipment, human resources, material resources, and acceptance of MHO principles. While this credentialing process limited the number of health facilities available to members, it ensured a minimum standard of care. Moreover, managers of private healthcare facilities, in particular, were motivated to improve their quality of care in order to meet these standards and attract new clients.

To encourage healthcare workers to provide superior services, a few MHOs offered bonuses. Others gave them small gifts as a token of appreciation for their collaboration.

*“Giving them 100 francs will not have the same value as the pen. Giving 1,200 francs will not have the same value as the T-shirt.*… *It changes the relationship”.* (Participant)

### Increase motivation of elected MHO members

The lack of motivation among elected members is a considerable constraint to enrolment and membership renewal. Elected members, responsible for recruiting new members and collecting fees, often complained that the workload is too much for unpaid work.

*“The man cannot leave his agricultural activities to devote himself to the MHOs. That would be difficult”.* (Participant)

MHOs were searching for ways to motivate elected members without changing the essence of voluntary work. Most promoters emphasized the importance of ensuring that elected members do not become accustomed to compensations that cannot be sustained once external funding ceases.

Various forms of financial compensation were adopted. Some promoters paid transportation costs. Others decided against it because such practice cannot be sustained once external funding ends. Other groups of MHOs offered elected members 4% to 10% of the membership fees they collected. This compensation was deducted from the MHOs’ revenues. Many participants reported that this had a positive effect on the amount of effort elected members were willing to dedicate.

*“We experimented with giving a fixed amount during the collection period but we realized that it’s not viable.… We needed an amount that is proportional to what the person collects”.* (Participant)

*“With the 10% we feel challenged. We are obliged to give more effort to get the 10%”.* (Participant)

A few promoters offered symbolic gifts to outstanding elected members. However, this initiative was recent and had been very minimally applied to date. Lastly, some promoters motivated elected members by providing paid training opportunities in different settings. Elected members called for more of these opportunities.

“*We are trying to develop opportunities to exchange experiences with other MHOs. For example, members from PROMUSAF can travel to work and exchange with members from CIDR and PISAF to see the mechanisms they have implemented there that we have not and that could reinforce our strategy*”. (Participant)

### Increase the level of satisfaction with health insurance coverage

Participants explained that low levels of enrolment and membership renewal are often due to people’s dissatisfaction with insurance packages. While the population’s needs and desires are systematically examined when MHOs are established, these evaluations are sometimes subsequently neglected, partly due to limited time and resources. Insurance products varied greatly among MHOs. Interviews with participants revealed that some MHOs covered uncommon but severe conditions, known as small risks, while others covered benign but more frequent conditions, known as big risks. Some participants reported that modifying an insurance package to include small risks had a positive effect on membership rates.

Some MHOs chose to omit valued health services such as prenatal consultations and ambulance services from insurance packages because they would require excessive membership fees. Participants explained that MHOs have to reach a compromise between their members’ demands and the additional costs resulting from more complete packages. When new health insurance needs emerge, technicians from the AIMS conceive different product options and determine their potential costs. To keep premiums low, technicians can turn to copayments or put ceilings on the reimbursement level. These different insurance products are discussed with the administrative committees of MHOs and adjusted as needed.

*“There are contradictions that arise where members are interested in having a product but they are not ready to make an effort to increase the membership fees. They are not ready to make the sacrifice.… We discuss the risk.… They have a choice to make.…”* (Participant)

MHOs affiliated with the RAS aimed to improve and diversify their insurance packages in order to meet people’s expectations and attract new members.

“*Our strategy is to always think of what we could propose that would fit with the needs of the population*”. (Participant)

These MHOs created two innovative types of collective insurance packages. The first of these, the “Student Insurance” plan, offered schools the possibility of simultaneously insuring all their students during school activities. As a collective package, it allowed MHOs to rapidly increase the number of beneficiaries. Moreover, MHO workers hoped it would inculcate values pertaining to health insurance in children, parents, and teachers, thereby creating more openness to these types of initiatives.

*“If we want tomorrow’s adults to have the notion of preparing for illness, we have to work on them now.… Second, we want to go through the children to touch the hearts of adults”.* (Participant)

Although this insurance package was perceived as highly effective in increasing the number of beneficiaries, participants reported some difficulties. First, the insurance product only covered illness occurring during school activities, since full-time coverage would have entailed higher fees, which many schools could not afford. Such limited coverage, however, could trigger undesirable effects. For instance, some parents may have felt their children’s health was now the school’s responsibility. In a context of poverty, some may even have been tempted to delay seeking medical care for their child during the weekend or summer so that the school’s insurance would cover medical charges. Moreover, participants highlighted that some parents might refuse to purchase family coverage under the pretext that their children were already covered by an MHO and they did not want to pay twice for health insurance. Another difficulty associated with the Student Insurance plan was that of convincing parents to support this school-based initiative and pay a premium for their children. Given that the government of Benin had made access to education free for all children, parents did not see why they should be asked to pay for this program.

*“Before, we could ask parents to contribute financially. Now, I can’t do that because the state said that school is free.… Most schools that accept are private schools”.* (Participant)

Another promising collective package, called “Maternity without Risks”, systematically covered all women in a village for prenatal medical consultations and health care received during birth. Village funds were created by requiring all citizens to contribute equally. Participants reported that the product was attractive because all women in the village, and therefore also their families, were confident they would eventually receive healthcare services covered by the MHO.

“*It helps people know about MHOs and it gives a better image*”. (Participant)

### Improve communication and information

*“In our culture, it is only when someone becomes sick that we ask the community to contribute financially to help a person*”. (Participant)

All participants reported that MHOs organized numerous activities to promote the importance of being prepared for illness and to increase people’s knowledge about the existence and benefits of MHOs. Some activities aimed to raise awareness through direct contact with community members.

*“We provided megaphones so that [elected members] can walk in the street and tell people it’s time to pay. It works well”.* (Participant)

On a weekly basis, elected members went door-to-door or gathered groups of people in public places to present the benefits of joining the plans. The large majority of participants considered door-to-door visits to be the most effective approach to convince people to join and to get members to pay their fees on time.

“*Going door-to-door, that’s what important. With radio, people listen without coming. But with door-to-door, if you meet someone and you talk to them, depending on your quality, they will enrol..*” (Participant)

Participants explained that the main limitations of door-to-door visits were that they required time, energy and dedication and incurred costs for elected members, who often had to travel to neighbouring villages by their own means.

“*It seems to be the strategy that results in collecting the most premiums. But it’s a strategy that requires resources! [We have to pay for] the gasoline to travel and so forth*”. (Participant)

Many MHOs recruited multiplying agents within their communities to promote new memberships and renewals. Some MHOs, for instance, collaborated with religious communities, schools, and political leaders. Beginning in 2005, some MHOs from the RAS created committees, each composed of a few villagers selected based on their social status and their commitment to developing MHOs in their communities. Their tasks were to share information, increase awareness, and act as resource persons when needed. They developed action plans and carried out promotional activities.

*“There are cases where the person is not educated but because of the place they occupy within the community, the position they have as community leader, religious leader or traditional leader, people listen when they talk.… When we don’t go through the person who is well listened to by the community, we often fail”.* (Participant)

PROMUSAF encouraged members to pay their premiums regularly by offering loyal members insecticide-treated bed nets at a low price. Selling these nets even at a modest price, rather than giving them away, established an ongoing source of revenue that would help sustain this strategy if ever the promoter were to withdraw its support and MHOs became entirely autonomous.

MHOs also attempted to raise awareness through mass communication campaigns, including radio broadcasts, theatrical plays, and village parties. The costs of these events represented a financial burden for MHOs. Participants had mixed opinions concerning the efficiency of theatrical plays, village parties, and dances.

*“We need to question ourselves concerning the efficacy of some activities.… Personally, I have reservations concerning theatre.… But I did see one case where traditional dancing and microphones really had an impact. The whole village danced. It created a good image”.* (Participant)

Participants agreed that radio broadcasting facilitated door-to-door activities by increasing knowledge about MHOs. However, they pointed out that mass communication generally did not, in itself, lead people to actually enrol. Thus, some participants highlighted the importance of carrying out awareness activities at both the individual and community levels.

PROMUSAF also trained its elected members to give educational sessions on subjects related to health and prevention. At these sessions, people were given preventive items, including prophylactics and insecticide-treated bed nets. According to one participant, there was a noticeable increase in enrolment after health education sessions.

*“We have observed that the day after we give educational sessions on malaria where insecticide-treated bed nets are given, there is an increase in enrolment in MHOs.… Over time, we realized that this is helpful and motivates people to join the MHOs”.* (Participant)

### Increase the level of trust in MHOs

The need to adopt strategies to increase the level of trust was reported in four of the 23 semi-structured interviews. These participants were affiliated with three different promoters, suggesting that this problem is not unique to a specific context. Some people wait before enrolling because they do not trust elected members and MHO managers.

*“They want to see whether the MHO is serious and whether it is managed well before they enrol”.* (Participant)

During communication activities, many MHOs presented testimonials of members who had previously received healthcare services covered by the MHO. Witnessing that MHOs had positive effects on others was reported to have increased people’s trust in MHOs.

“*This allows people to understand that this initiative is real!*” (Participant)

Trust is also built on democratic management. Members are encouraged to select leaders based on the candidates’ honesty. Transparency is also promoted by hosting a general assembly where financial statements are presented.

### Reinforce governmental involvement

Efforts to involve local authorities varied among the different MHO promoters. One promoter representative reported having been hesitant to develop strong partnerships with political leaders at first.

*“We have to leave a separation between politics and the MHO. There are risks of political profiteering.… That’s why we waited before we approached these decision levels”.* (Participant)

Most promoters reported that they systematically tried to involve local authorities in the long-term development of MHOs.

*“We recommend establishing a start-up committee, with the involvement of elected leaders from each district, to drive the project of creating an MHO.… After they are created, we have exchange workshops with local leaders to discuss their development.… Then we help them establish what we call a local mechanism of continuing support to MHOs.…”* (Participant)

In some instances, local leaders have displayed strong political will by helping to finance insurance packages. However, mobilizing political leaders remains a challenge.

“*The first difficulty is to convince elected leaders and to help them understand the importance of their involvement”.* (Participant)

“*Some people think that [the MHO] is free money that has come in….When they feel they aren’t gaining anything, they refuse”.* (Participant)

PISAF contributed, both financially and technically, to the creation of a *Strategic Plan to Develop MHOs 2007–2011* for the government, which aimed to promote the development of MHOs across Benin. Among other things, the document proposed adopting a legal framework for MHOs. At the time of the interviews, participants reported that the document was in the process of being validated by the Ministry of Health.

*“It seems like [the document] is going in the right direction…but when we say in the right direction, in our country, we know what that means! It could take two, three, four years and still nothing comes out”.* (Participant)

Overall, participants called for more governmental involvement. Many of them believed that, ultimately, the development of MHOs will require a legal obligation for people to be covered by health insurance.

“*For me, the solution is that [health insurance] becomes obligatory and that there’s a real constraint to enroll. Without this, MHOs will not survive”.* (Participant)

## Discussion

Research has shown that MHO subscription has positive outcomes on members. In Benin, MHO members report less difficulty in accessing care, lower out-of-pocket expenditures, and greater empowerment, especially with respect to healthcare workers’ abuse of power [22,23]. On the other hand, MHOs’ success has been limited by a low penetration level. Scientific evidence on the causes of low membership rates is well established [24]. The current knowledge gap pertains to the strategies MHOs have adopted to overcome these factors. This is the first study to map and review MHO initiatives aiming to increase coverage of the target population. Using a multiple case study design, the present study contributes new information and insights that can inform MHO actors across West Africa. More specifically, the results:

• Provide an overview of the scope and range of actions developed and implemented in Benin to target entry points that influence membership, including payment modalities, motivation of elected members, quality of healthcare, satisfaction with coverage, population trust, communication and information, and government involvement.

•Suggest that optimal approaches must be comprehensive and inclusive, as no single MHO initiative has been able to resolve the issue of low membership on its own.

•Reveal that going door-to-door, a relatively simple and low-cost initiative, is considered to be the most effective method to increase membership, while other complex initiatives, such as theatrical plays and dances, are considered less effective.

•Highlight that verbal agreements between MHOs and healthcare providers are not sufficient to foster good relations and quality of care. Tighter partnerships with healthcare providers are necessary, with opportunities for dialogue and mechanisms to prevent and resolve conflicts.

•Indicate that the creation of collective insurance products for specific subgroups, such as students and pregnant women, is considered a highly promising avenue to increase MHO membership.

•Show that securing commitment and investment from political leaders remains a challenge despite efforts by some MHOs to establish durable partnerships early on.

•Show how the progress of numerous initiatives is limited by the financial, material and human resources available.

Overall, the results highlight the importance of adopting sustainable strategies to promote the development of MHOs. Projects funded by international cooperation agencies generally have limited time periods. In Benin, the majority of MHO promoters plan on gradually withdrawing their support to allow the MHO movement to become autonomous. In some cases, the promoters have already initiated this transition process. If MHOs are to continue to grow after promoters retreat, they must rely on strategies that are feasible within their own financial and technical means, particularly in a context where government support is uncertain. Participants agreed that ending effective but unsustainable practices to which members, workers or healthcare providers have become accustomed could significantly harm MHOs in the long term. Promoters must prepare MHOs for independence early on by continuously engaging in knowledge transfer and capacity reinforcement.

The results indicate that MHOs have established different types of payment modalities to influence decisions to enrol. Family membership with premiums varying according to family size may be advantageous for MHOs by reducing adverse selection and making it easier for larger families to join [5,25]. However, De Allegri [26] stressed the importance of adopting a definition of household that suits the reality of everyday decision-making. In a study on consumer preferences, participants requested that MHOs allow smaller family units to join to reflect their economic reality [11]. Payment modalities should be selected in line with the target population’s needs and socioeconomic environment [5].

The results suggest that payments in kind have facilitated the ability to pay premiums in rural settings. Webber [27] argues that MHOs can adopt payments in kind if the goods traded are homogeneous and, easy to store and transport, and if the MHO can easily sell the goods.

The results highlighted the importance of aligning premium collection with the availability of financial resources. Annual or bi-annual premiums should be adopted for agricultural workers who have an influx of revenue once or twice per year [28]. In contrast, monthly premiums should be used for salaried workers [28]. Payment periodicity has repercussions on the volunteers’ workload. The availability of a central location where premiums can be dropped off, such as a local store, could facilitate frequent premium collection periods. Moreover, monthly premiums require more work because the list of members in good standing must be updated and sent to healthcare facilities each month. Failure to update this list can result in MHOs paying healthcare services for individuals who have not renewed their membership. Overall, best practice requires that MHOs explore community preferences and strike a balance between the target population’s contributive capacity and the MHOs’ internal resources.

Low healthcare quality is recognized as one of the most important constraints to enrolment and membership renewal [9]. In addition to frequent drug shortages, MHO members often must endure the negative behaviour of healthcare providers. Devising effective ways to overcome such fundamental deterrents to membership is urgent. Although few effective solutions have yet been found to prevent medication shortages, there is some hope for improving behaviours. Our results revealed that closer partnerships between MHOs and healthcare providers can improve dialogue and interactions, especially if both parties perceive the relationship as profitable. Key elements of this process can include providing medical advisors and continuous training and supervision for healthcare workers [29,30]. Letourmy [29] observed that contracts with clauses on quality of service and availability of medication were useful in Mali, an approach corroborated by our results.

There is little research on how to motivate volunteers managing MHOs in West Africa. Long-term volunteers can experience boredom, tiredness, resentment over loss of time, and frustration. According to Haski-Leventhal and Bargal [31], events such as the arrival of new and motivated volunteers, the development of new operating methods, and the adoption of new roles might counteract boredom and tiredness. Our results suggest that elected members are calling for greater financial compensation. Yet, Deci and Flaste explain that providing financial compensations may undermine intrinsic motivation, causing volunteers to focus on the compensation rather than on the organization’s mission in itself [32]. Introducing compensations proportional to the number of recruits could even lead members to manipulate and distort information in order to persuade non-members to join [32]. Evidence is needed on how to enhance elected members’ intrinsic motivation to promote MHOs without threatening the MHOs’ integrity or financial sustainability.

Our results show that collective insurance packages targeting elementary schools, work associations, and pregnant women in villages are beginning to emerge in Benin. Participants described these trials as promising. Supporting this view, Wipf and colleagues [33] explained that targeting health insurance to pre-existing groups brings in lower-risk individuals who would otherwise wait until they were older or at greater risk of illness before enrolling. By enabling MHOs to reach high volumes rapidly, collective insurance lowers collection costs and time. Wipf and colleagues [33] argue that group coverage can cost half as much as individual insurance because higher sales reduce the unit costs of underwriting, administration, and claims. However, experiences in countries like Uganda show that insurance plans based on mandatory coverage, such as group insurance, sometimes take their “captive” members for granted and make little effort to provide information to consumers. It is imperative that MHOs continue marketing and education efforts to constantly promote the values of health insurance, especially when people are obliged to purchase an unsought product [33]. Although participants in the present study called for more systematic enrolment mechanisms, there is a need to test whether the organizational capacity will be sufficient in the long run to sustain this approach in everyday practice and different settings.

An important issue stems from the potential conflicts between collective and family insurance products. There is a risk that some families, for instance, may not want to enrol or renew their membership if their children have access to health insurance at school. At the time of the study, MHO stakeholders were developing collective and family packages offering different coverage to avoid overlaps. Efforts were being devoted to raising awareness about the benefits of having complementary coverage. Good communication is central to increasing social acceptability of these complementary products. However, as the availability of collective health insurance products increases in diverse settings, MHOs may have no choice but to develop mechanisms to allow members to withdraw from overlapping insurance coverage.

Our results show that MHOs combined different types of mass communication with personal communication to increase membership. According to Rogers [34], mass media channels are the most rapid and efficient means of informing individuals about the existence of an innovation. On the other hand, interpersonal channels are more effective in changing attitudes toward a new idea, particularly if the communication is conducted between similar individuals. Communication between individuals sharing personal and social characteristics often leads to greater attitude formation and behavior change [34]. This explains why participants in the present study found door-to-door activities with elected MHO members to be the most effective initiative to increase enrolment and membership renewal. It also supports the MHOs’ current practice of sharing testimonials of MHO members who successfully received healthcare. To ensure effective communication, MHOs should select leaders and multiplying agents who are representative of different subgroups of the population.

Rogers [34] explains that social innovations are diffused in society by reaching different categories of consumers at different speeds, from the most enthusiastic to the most reluctant. The main challenge is to transition from an innovative type of clientele to mass diffusion. Earlier and later consumers of innovations have distinctive characteristics in terms of socioeconomic status, personality variables and communication behaviors. Thus, these consumer types can be used for audience segmentation, that is, using different communication channels or messages to reach different subgroups. For example, early adopters (innovators) are more likely to be influenced by mass media channels and scientific research by experts. In contrast, later adopters tend to rely on interpersonal channels such as subjective evaluation of peers. Rogers [34] points out that each type of consumer must pass through a decision process going from (1) knowledge of an innovation, to (2) persuasion, (3) decision to adopt or reject, (4) implementation, and (5) confirmation of the decision. Different communication sources or channels work best at different stages of this process [34]. This current of thought suggests that MHOs need to overcome resistance by tailoring their channels to each consumer type and each adoption stage to increase membership.

Participants in this study reported the need to build trust in MHOs by sharing successful testimonials, relying on democratic management and ensuring transparency. Nevertheless, Ridde et al. [23] found that both members and non-members already have a strong sense of trust in MHOs. Together, these studies suggest that establishing trust in MHOs may be necessary but not always sufficient to increase membership. We recommend that MHO stakeholders evaluate the community’s perception of MHOs on a case-by-case basis and adopt context-specific strategies.

Our findings suggest that health education workshops and insecticide-treated bed nets increase enrolment. According to Radermacher and colleagues [35] such preventive health activities may be beneficial to maintain ongoing communication with the community and to provide all members with a tangible benefit, even those who do not use healthcare services. Similarly, Microcare, a microinsurance organization in Uganda, offered HIV prevention activities and a malaria prevention program that distributed insecticide-treated bed nets. In Bangladesh, microinsurances organized annual health check-up camps for their members. By preventing diseases, such activities may lower the financial burden of illnesses that cause high insurances costs [35]. Empirical assessments of their cost-effectiveness are warranted.

Although Criel and Walkens [9] found that some communities were open to the idea of exempting the poor from membership fees, our results show that this policy had generally not been adopted by MHOs. In most cases, MHO decision-makers, even if favourable to exemptions, do not have the financial means to carry out this practice without putting MHOs at risk of bankruptcy. In this situation, MHO decision-makers have been found to prioritize financial viability over solidarity [36]. Additional government funding would be useful to reduce premium fees for the poor or destitute.

Participants described the government of Benin’s support as largely insufficient. In Uganda, Basaza and colleagues observed poor knowledge and understanding of MHO activities among government officials [37]. Two-thirds of the Ministry of Health staff and one-fifth of the health district officers could not name more than two characteristics of MHOs even after probing. Thus, MHOs in Benin should join forces to develop an organized plan to expand the government’s understanding of MHO concepts and functioning. Means of communication with government officials can include workshops, seminars, university studies, reports from the MHOs, and media [37]. In a review of the role of states in promoting microinsurance, Ranson and Bennett [38] explained that support can include policy frameworks, subsidies to pay premiums for the poor, direct financial support, drugs, equipment, staff, and access to facilities.

A key question is whether it is possible to reach high coverage levels without active government support. The World Health Report [1] states that multiple health insurance pools serving different population groups are inefficient and that small pools are not viable. The report also suggests that it is impossible to achieve universal health insurance coverage when enrolment is voluntary. Two countries, Ghana and Rwanda, have demonstrated how strong political commitment and investment can rapidly increase membership [39-42]. Both governments passed laws making health insurance obligatory. They also designed universal health insurance regimes (UHIR) that fully integrated MHOs into their structure. In Ghana, 67% of the population was covered six years after implementation of its compulsory National Health Insurance System [42]. In 2010, MHOs in Rwanda covered 80% of the population [1]. We think, however, that factors specific to Rwanda’s contexts, such as the high population density and the more authoritative political regime, may have facilitated the implementation of these policies.

There is a risk that MHOs financially tied to governments may experience less autonomy and liberty of action. Ranson and Bennett [38] emphasized the need for caution, as even well-intentioned government support can change the nature of MHOs, thereby undermining transparency and community ownership. In order to grow, MHOs will have to balance between effectiveness and financial robustness, on one hand, and transparency and community ownership on the other.

The government of Benin has announced it will launch a UHIR [43,44]. Thus far, however, MHOs’ involvement in the development of the UHIR has been weak [43]. The challenge for MHOs is to be recognized as a relevant actor in both the conceptualization and the implementation of this policy. Intensive lobbying of government authorities should be considered. A united front of MHO actors may be the best vehicle for this enterprise.

Little evidence exists on the implementation processes and ensuing outcomes of interventions to increase MHO membership in West Africa. There is an urgent need to set up more mechanisms to facilitate information exchange and structural relations between MHOs. In these circumstances, we recommend the compilation of a repertoire of strategies for increasing enrolment and membership renewal. This would be useful to compare the performance of different initiatives, to attribute effects to specific mechanisms, and to guide decision-making. It would also facilitate future research and allow MHO promoters to improve inter-organizational dialogue.

The need to promote dialogue between MHOs led to the creation of La Concertation (http://www.concertation.org) in 1999. This structure hosts international conferences to promote dialogue among MHO actors and conducts an inventory of MHOs every three years. Participants in the present study did not discuss the strategic roles of this organization, so we were unable to draw any conclusions regarding how it is assessed in the field. In the future, La Concertation could play an important role in creating a repertoire of strategies to increase MHO coverage in West Africa.

A review of past experiences in Mali, Senegal, and Guinea suggests that MHOs can benefit from banding together as unions or federations [45]. In addition to promoting exchange of knowledge and experiences, MHO networks may facilitate advocacy and lobbying aimed at influencing political authorities. By combining pools of members, networks also increase risk sharing, leading to economies of scale and financial stability [45]. Activities and services that would not be accessible to a single MHO can be developed. Networks also give MHOs more leverage to negotiate drug availability and quality of healthcare with providers [29]. Future studies should examine the strengths and limitations of different types of MHO networks [45].

### Limitations

Past studies have shown high consistency with regard to the factors that lower MHO enrolment and membership renewal. Therefore, we are confident that the initiatives and lessons learned reported in this multiple-case study are relevant for other African contexts. Before generalizing across regions, however, there is a need for more solution-driven research to empirically evaluate the implementation processes, efficiencies, and costs of the different initiatives and partnerships. The effectiveness of any strategy is contingent upon the specificities of the environment. MHO representatives should consult their population regarding their needs and preferences in order to adopt appropriate initiatives.

One limitation of the study lies in the sample of interviewees. While an effort was made to recruit participants from different settings and origins, MHOs in deep rural areas are somewhat underrepresented. A second limitation is the potential existence of a social desirability bias. We tried to minimize this by explaining the strict separation between the study and any MHO and by avoiding the presence of other MHO representatives at interviews. The high level of concordance among interviewees and their openness about the limitations of the different initiatives suggest that the social desirability bias was fairly well controlled.

## Conclusion

The majority of MHOs in West Africa are characterized by low membership levels that threaten their long-term viability. The present study provides a better understanding of initiatives adopted by MHOs to counteract factors that hamper enrolment and membership renewal. Our results highlight the wide range and scope of initiatives targeting different entry points and involving various stakeholders. The diversity of experiences illustrates MHOs’ inability to find a single solution to increase membership. Ultimately, and according to participants, the solution probably lies in a UHIR that incorporates MHOs into its structure. Until such a regime is fully implemented in Benin, the survival of MHOs is tied to their capacity to attract the population. In a context of voluntary enrolment, MHOs may benefit from adopting comprehensive approaches that target entry points such as payment modalities, motivation of elected members, quality of healthcare, relationship between patients and healthcare workers, sense of satisfaction with coverage, trust in MHOs, communication and information, and government involvement. MHOs may also benefit from adopting inclusive approaches that lead to concerted actions with key stakeholders such as household decision-makers, elected members, healthcare workers, medical advisors, local leaders, government authorities, and promoters. More dialogue between MHO representatives would be useful to devise innovative strategies, avoid repeating unsuccessful ones, and develop an organized plan to promote MHOs in the communities.

## Appendix A

### Guide for Semi-structured Interviews

1. How would you qualify MHO membership? Do these results correspond with your expectations?

2. According to you, what are the constraints that affect enrolment in MHOs?

3. What strategies have been adopted to counteract these constraints and increase the enrolment of the target population?

4. According to you, what are the constraints that affect renewal of MHO membership?

5. What strategies have been adopted to counteract these constraints and increase membership renewal?

6. Can you describe the process that led to the development of strategies for increasing membership?

7. How did you select the strategies that were adopted?

8. How did the implementation of these strategies go? How did MHO members react?

9. How did these strategies affect the membership in comparison to your expectations?

10. How do you envision MHOs in ten years?

## Competing interests

The authors declare that they have no competing interests.

## Authors’ contributions

AMTT designed the study, collected and analyzed data, and drafted the manuscript. SH designed the study and reviewed the manuscript. IY designed the study, helped with data collection and reviewed the manuscript. PF designed the study and reviewed the manuscript. All authors read and approved the final manuscript.

## Supplementary Material

Additional file 1 **Table S2.** Repertoire of Initiatives to Increase Enrolment and Membership Renewal in Benin. Click here for file
