# Supplementary material for: Mapping of initiatives to increase membership in mutual health organizations in Benin
Source: Int J Equity Health. 2012 Dec 5;11:74. doi: 10.1186/1475-9276-11-74 (PMC3541096; doi:10.1186/1475-9276-11-74)
Supplement: Additional file 1 — Table S2. Repertoire of Initiatives to Increase Enrolment and Membership Renewal in Benin. [file 1475-9276-11-74-S1.doc]

**Table S2.** Mapping of Initiatives to Increase Membership in MHOs

| **Initiatives** | **Example of Initiatives** | - **Advantages** - **Difficulties** |
| --- | --- | --- |
| **Facilitate payment of premiums** | Accept premiums in the form of crops | - Allows people to pay their membership when there is a lack of cash - Requires a large and safe storage area - Market price can decline before crops are sold |
|  | Adapt payment modalities to people’s needs (monthly vs. annual premiums) | Monthly:   - Allows progressive payments when members’ income is monthly - Highly time-consuming - Difficult to keep track of members’ status   Annual:   - Adapted to the periods of revenues in rural areas - Less time-consuming to collect premiums |
|  | Adapt membership levels to people’s needs (e.g. individual vs. family) | Individual:   - Adapted to the individualism emerging in cities - Reduces risk-sharing   Family:   - Easier for large families to enrol when the price depends on the family size - Efficacy depends on the level of community solidarity |

pr

|  | Organize revenue-generating activities, such as collective fields, to lower premiums | - External funding possibly needed because MHO resources are limited |
| --- | --- | --- |
| **Increase satisfaction with health insurance coverage** | Evaluate populations’ needs and expectations | - Evaluation neglected because of limited time and resources - Broadening coverage incurs greater premium fees |
|  | Create collective packages (e.g. “Student Insurance” and “Maternity without Risks”) | - Rapidly increases the number of beneficiaries - Promotes values pertaining to health insurance in new subgroups of the population - Collection of funds is done by the group leaders - Facilitates membership renewal - Coverage is adapted to needs of the subgroup - Limited coverage is offered to keep premiums low |
| **Improve healthcare and the patient–healthcare worker interpersonal relationship** | Contract with healthcare centres | - Prevents conflicts - Requires medical advisor to negotiate with administration |
|  | Work in partnership with a network of health care centres (e.g., “Network for Coordinated Care”) | - Helps coordinate and standardize health services - Establishes ongoingdialogue between healthcare centres and MHOs - Leads to progressive improvements in healthcare workers’ attitudes |
|  | Involve healthcare workers in the MHOs’ development (collaborative approach) | - Increases the credibility of MHOs - Gradually improves the relationship with healthcare workers - High risk of corruption among healthcare workers (e.g. illegitimate prescription of medication) |
|  | Develop MHOs and healthcare centres simultaneously (co-development approach) | - Gives healthcare workers a good perception of MHOs - Cannot be sustained by MHOs (requires external funding) |
|  | Obtain support from a medical advisor | - Medical knowledge facilitates negotiations with healthcare workers - Demand cannot be met due to costs |
|  | Provide training to healthcare workers | - Improves understanding of MHOs - High level of mobility among healthcare workers (training must be repeated often as new nurses are hired) |
|  | Carefully evaluate and select healthcare centres | - Ensures a minimum standard of care - May motivate private healthcare centres to improve their quality of care to receive MHO clients |
|  | Offer small bonuses and symbolic gifts to healthcare workers | - Appreciated by healthcare workers - Must not undermine their intrinsic motivation - Must be sustainable - Limited by resource constraints |
|  | Ask vendors close to healthcare centres to act as information booths on MHOs; provide them with basic training in MHO principles so that they can act as resource person during conflicts | - Ensures that an MHO representative is always available - Less work for elected members - Increases MHO visibility |
|  | Request that healthcare workers wear name tags | - Low cost - Ensures that health care agents can be traced if they act inappropriately |
|  | Remind MHO members of their rights and obligations | - Can prevent conflicts |
| **Improve communication and information** | Increase awareness through direct contact (e.g. megaphones, door-to-door visits, multiplying agents, promotion of prophylaxis) | - Effective - Requires significant time and energy from elected members - Can incur costs for elected members |
|  | Conduct mass communication campaigns (e.g. radio, theatre) | - Can improve passive awareness - Limited efficiency due to high costs |

| **Increase trust in MHOs** | Share testimonials | - Allows others to see that MHOs can have positive impacts |
| --- | --- | --- |
|  | Elect members whom people trust | - Members do not always elect the most honest and competent candidates |
|  | Promote transparency (e.g. present financial statements) | - Corruption persists within some MHOs |
|  | Promote good administration (e.g. provide training for elected members) | - Low education level of elected members can limit their capacity to run MHOs - Capacity building requires external assistance |
| **Reinforce governmental involvement** | Seek the support of authorities to implement and finance MHOs | - Must increase awareness among local leaders - Some leaders may be interested in hopes of accessing funds |
|  | Work with the government to develop national strategies | - Requires external funding - Processes slowed by bureaucracy |
| **Increase elected members’ motivation** | Provide compensation for transportation | - Compensation must be sustainable - Compensation must reflect the efforts invested |
|  | Provide symbolic gifts | - Compensation must be sustainable |
|  | Provide training | - High costs |
